# Effects of Feed Composition in Different Growth Stages on Rumen Fermentation and Microbial Diversity of Hanwoo Steers

**DOI:** 10.3390/ani12192606

**Published:** 2022-09-28

**Authors:** Chae Hwa Ryu, Han Tae Bang, Seul Lee, Byeonghyeon Kim, Youl Chang Baek

**Affiliations:** Animal Nutrition and Physiology Division, National Institute of Animal Science, Rural Development Administration, Wanju 55365, Korea

**Keywords:** feed composition, growth stages, rumen fermentation, microbial diversity, Hanwoo steers

## Abstract

**Simple Summary:**

Ruminants are a major source of greenhouse gases and environmental pollution, which are exacerbated by excessive feeding because excess nutrients are excreted. Since digestion in ruminants is aided by both microorganisms and digestive enzymes, information on gut microbiota in ruminants is vital for assessing the adequate amount of feed supply. The growth of Hanwoo steer is divided into three stages, and specific nutrients are provided in each stage. Growth stages are a major factor influencing rumen fermentation by microorganisms. In this study, changes in fermentation characteristics and microbial community during the growth stages were investigated. Our findings show that there were differences in the levels of total volatile fatty acids, propionate, and valerate. In addition, we found that the relative content of some bacteria and fungi changed with the growth stage. This study improves our understanding of rumen fermentation and changes in the gut microbiota during the various growth stages of Hanwoo steer.

**Abstract:**

Ruminants are a major source of greenhouse gas emissions, and information on ruminant fermentation and microorganisms is essential to understand ruminant digestion, which is associated with environmental pollution. The present study investigated rumen fermentation and microbial diversity according to the three different growth stages of four Hanwoo steers: growing (12 months, G), early fattening (18 months, EF), and late fattening (25 months, LF). No significant differences were observed in rumen pH and ammonia nitrogen among growth stages. Total volatile fatty acids were significantly higher and propionate and valerate significantly lower in G than in EF and LF (*p* < 0.05). Ten bacterial phyla were detected, including Firmicutes (47.5–53.5%) and Bacteroidetes (28.4–31.7%), which accounted for 79.2–82.3% of the total bacteria. Prevotella accounted for the highest proportion (31.6–42.6%) of all bacteria in this study but did not differ significantly among the different growth stages. Metaprevotella abundance was significantly higher in G than in the other treatments (*p* < 0.05). In addition, Paraprevotella tended to be higher in LF than in the other treatments (*p* = 0.056). Given the differences in the genera of microorganisms with relatively low abundance, additional experiments are needed to determine the effect on fermentation.

## 1. Introduction

Ruminants are a major source of greenhouse gas emissions globally. The amount of nutrients supplied to Hanwoo steers is based on productivity; however, such practices should be reconciled with environmental issues such as nitrogen emissions. This is because the excessive ingestion of nutrients reduces utilization efficiency and causes environmental pollution when excreted in feces and urine [1].

The growth of Hanwoo steers is divided into three stages, and the recommended nutrient levels differ for each growth stage. The organs and body tissues of Hanwoo grow between 6 and 12 months of age, and red meat increases until approximately 20 months of age [2]. Marbling increases between 12 and 25 months, affecting meat quality [2]. Most of the feed sold in South Korea is designed to increase both the forage ratio and crude protein (CP) in concentrates for the development of the rumen during the growing stage. As the fattening stage progresses, the forage ratio is gradually reduced and the total digestible nutrients in concentrates are increased to provide a high energy source.

The nutrient digestion process of ruminants differs from that of monogastric animals in that decomposition occurs not only through digestive enzymes but also through microorganisms living in the rumen [3]. Ruminant microorganisms can decompose and synthesize nutrients simultaneously [4]. Therefore, this nutrient decomposition process directly affects the maintenance of physiological functions and maximization of ruminant productivity [5]. However, researchers have not been able to obtain accurate results on the nutrient digestion process in ruminants owing to the lack of information on ruminant microorganisms, limited research conditions, and process errors [6].

Understanding the composition and content of rumen microbes is essential for predicting rumen fermentation properties and animal health [7,8]. Feed affects the rumen microbiome by increasing or decreasing the microbial population [9]. Many studies have been conducted to determine the interaction between rumen fermentation and microorganisms [9,10,11]. However, previous studies mostly relied on anaerobic culture methods, and information on rumen microbial diversity remains limited. Although the rumen microbiome plays an important role in ruminants, information on how the rumen microbiome improves fermentation or digestibility and reduces methane is limited. Therefore, this study investigated the effects of different growth stages on the rumen fermentation characteristics and microbial community of Hanwoo steers.

## 2. Materials and Methods

### 2.1. Animals, Diets, and Experimental Design

The subjects in this experiment were four Hanwoo steers from the National Institute of Animal Science who underwent all growth stages. The animals were fed at least 6 months in each growth stage and used to collect rumen samples after acclimatization. The growth stages were: growing (G, 12 months, 333.4 ± 4.38 kg of body weight (BW)), early fattening (EF, 18 months, 538.0 ± 16.98 kg of BW), and late fattening (LF, 25 months, 651.6 ± 22.25 kg of BW). Diets per growth stage were provided according to the Korean Feeding Standards for Hanwoo: timothy grass and concentrate for G (4:6), grass hay and concentrate for EF (2:8), and rice straw and concentrate for LF (1:9). Steers were fed 1.5% of their BW twice daily (at 800 and 1600) as forage and commercial concentrate (Woosung Co., Daejeon, Korea). Fresh water and mineral blocks were available *ad libitum* throughout the experimental period.

### 2.2. Chemical Analysis of Experimental Feed

All feed samples used in the experiment were dried at 60 °C for 48 h and ground in a cyclone mill (Foss, Hillerød, Denmark) fitted with a 1 mm screen. Dry matter (930.15), acid detergent fiber (973.18), ash (942.05), and ether extract (EE; 2003.05) were analyzed using the procedure reported by Horwitz and Latimer [12]. Neutral detergent fiber (NDF) was analyzed using a heat-stable amylase and expressed inclusive of residual ash (aNDF) [13]. CP was calculated as 6.25 times the nitrogen content, and total nitrogen was measured via the Dumas combustion method using an elemental combustor (Vario Max Cube, Elementar Gmbh, Frankfurt, Germany). The acid detergent-insoluble CP and neutral detergent-insoluble CP (NDICP) levels in each sample were determined according to the method described by Licitra et al. [14]. Non-fiber carbohydrates (NFC) were calculated as 100-ash-EE-CP-(aNDF-NDICP) based on the guidelines provided by the National Research Council (NRC) [15]. The experimental feed is described in Table 1. 

### 2.3. Rumen Fermentation

Rumen fluid (three replicates in each group) was collected using a stomach tube 2 h after each morning feed as described by Duffield et al. [16]. The pH of the rumen fluid was determined immediately after collection using a general-purpose pH meter (EcoMet P25, Istek, Inc., Seoul, Korea), and then, the rumen fluid was transferred to the laboratory. The volatile fatty acid (VFA) concentration was determined as described by Erwin et al. [17]. Briefly, 1 mL of the rumen fluid supernatant was mixed with 0.2 mL of 25% (*w*/*v*) metaphosphoric acid and kept at 4 °C for 30 min. After centrifugation of the mixture at 13,000× *g* for 10 min at 4 °C, the supernatant was injected into a gas chromatograph (HP 6890, Hewlett-Packard CO., Palo Alto, CA, USA) equipped with a flame ionization detector and capillary column (Nukol fused silica capillary column 30 m × 0.25 mm × 0.25 μm, Supelco, Inc., Bellefonte, PA, USA). The temperatures of the oven, injector, and detector were 180 °C, 220 °C, and 200 °C, respectively. The ammonia nitrogen concentration was determined using the method described by Chaney and Marbach [18]. Briefly, 0.02 mL of culture supernatant was mixed with 1 mL of phenol color regent and 1 mL of alkali-hypochlorite reagent and then incubated at 37 °C in a water bath for 15 min. Then, the optical density was determined at 630 nm using a spectrophotometer (Optizen Pop, Mecasis, Korea).

### 2.4. Deoxyribonucleic Acids Extraction

DNA was extracted from rumen fluid samples using a repeated bead beating plus column method [19]. In each 0.25 g rumen fluid sample, 1 mL of lysis buffer (500 mM NaCl, 50 mM Tris-HCl, pH 8.0, 50 mM EDTA, 4% sodium dodecyl sulfate) and sterile 0.4 g of zirconia beads (0.3 g of 0.1 mm and 0.1 g of 0.5 mm) were added and homogenized for 3 min at the maximum speed on a Mini-Beadbeater^TM^ (BioSpec Products, Bartlesville, OK, USA). The tubes were then incubated at 70 °C for 15 min with gentle shaking by hand every 5 min. This was followed by centrifugation at 4 °C for 5 min at 16,000× *g* and the addition of 300 μL of lysis buffer. After centrifugation (16,000× *g* for 5 min at 4 °C), the supernatant was mixed with 260 μL of 10 M ammonium acetate, and incubated on ice for 5 min. After centrifugation (16,000× *g* for 5 min at 4 °C), the supernatant was mixed with 1 volume of isopropanol, the nucleic acid pellet was washed in 100 μL of Tris-EDTA buffer, and the two aliquots were pooled. The precipitated nucleic acids were then treated with RNase A and proteinase K, and the DNA was purified using columns from the QIAgen DNA Mini Stool Kit (QIAGEN, Valencia, CA, USA).

### 2.5. Rumen Microbial Diversity

The amplicon library of the 16S/18S rRNA gene was prepared from each composite DNA sample using specific primers (Table 2). Paired-end sequencing was carried out on a MiSeq platform (Illumina, San Diego, CA, USA). Paired-end reads were merged with a minimal overlap using the FLASH program [20]. The bioinformatic programs in the QIIME software package (version 1.9.1) were used for all sequence processing and analysis [21]. The resulting files were divided into classes of species identified using BLASTn (version 2.4.0) compared to a curated high-quality 16S/18S database derived from the National Center for Biotechnology Information (NCBI). Operational taxonomic units (OTUs) were determined using a similarity threshold level of 97% between sequences to classify microorganisms at the species level. Data were compiled, and relative percentages were determined for each individual sample. Alpha diversity indices, such as OTUs, Chao1 (richness), Shannon (diversity), Gini-Simpson, and Good’s coverage, were calculated based on a UniFrac matrix. 

### 2.6. Statistical Analysis

Data for rumen parameters and the relative quantities of rumen microbial species were analyzed using analysis of variance of a general linear model in SPSS (Version 18, IBM, New York, NY, USA). Differences among means were compared using Duncan’s multiple range test when there was a significant overall effect. Significance was set at *p* < 0.05, and 0.05 ≤ *p* < 0.1 was identified as a trend.

## 3. Results and Discussion

Rumen fermentation parameters from each treatment are presented in Table 3. The rumen pH was within the appropriate range of 5.8 to 7.2 [26]. All samples maintained a pH ranging from 5.93 to 6.35. Ammonia nitrogen production is an indicator of the decomposition and synthesis of proteins by rumen microorganisms. A minimum of 5–8 mg/dL and a maximum of 29 mg/dL are produced in the rumen [27]. This production was found to range from 19.12 to 23.97 mg/dL in all growth stages, and there was no significant difference among growth stages. Total VFA (TVFA) was significantly higher in G than in the other growth stages (*p* < 0.05). Calabro et al. [28] reported that the higher the NDF digestibility, the higher the TVFA production. The NDF digestibility predicted based on the chemical composition of feed and the digestibility formula provided by the NRC [15] was observed to be higher in G than in the other growth stages, which is thought to have affected the results. Propionate and valerate were significantly lower in G than in EF and LF (*p* < 0.05). When the forage feed amount was high, the acetate to propionate ratio was high because acetate was high and propionate was low. Fast-acting compounds such as starch and sugar are digested in the rumen two hours after feeding. Therefore, in the experimental group with high concentrated feed, it may affect propionate, especially among fermentation properties. The proportion of propionate was significantly higher in the EF and LF than in G. The acetate to propionate ratio in G with a high forage ratio was significantly higher than that in the other growth stages (*p* < 0.05), showing consistent findings with that of previous studies [29,30].

**Figure 1:**
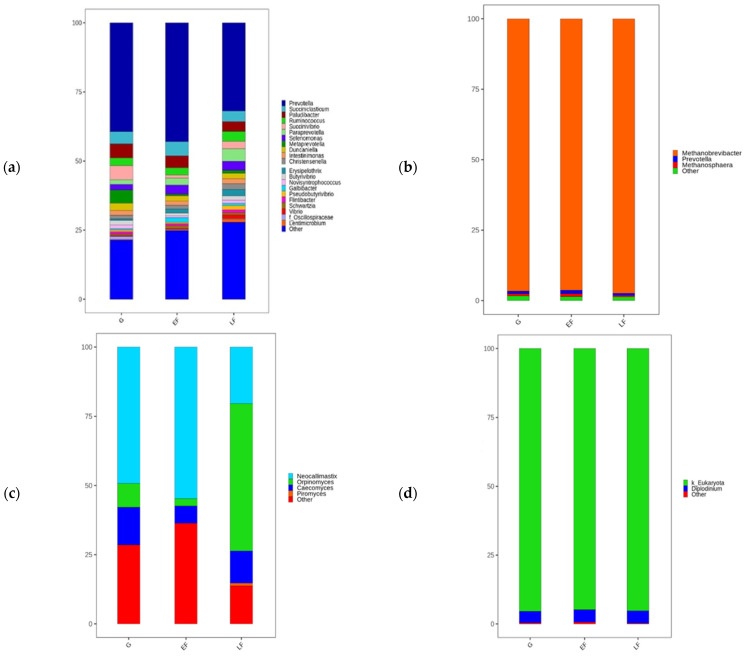
Relative abundance of rumen microbial diversity for the genus level from Hanwoo steers at different growth stages. Relative abundances of (**a**) bacterial genera, (**b**) archaeal genera, (**c**) fungal genera, and (**d**) protozoan genera are shown.

The number and diversity of microorganisms in the rumen were measured based on OTUs, and the Shannon, Chao 1, and Gini-Simpson indices, and the results are shown in Table 4. The accuracy levels of species diversity measurements were as high as 99.6 to 100.0%, but no significant differences were observed among growth stages. The effects of different growth stages of rumen microbial diversity are shown in Figure 1. The sequence reads of microorganisms were isolated as bacteria (1,005,325), archaea (895,500), fungi (841,038), and protozoa (969,350) when the nucleotide sequence accuracy exceeded 95%. A total of 25 microorganisms identified at the phylum level were observed in all samples regardless of growth stage. A total of ten bacterial phyla were detected, including Firmicutes (47.5–53.5%) and Bacteroidetes (28.4–31.7%), which accounted for 79.2–82.3% of the total bacteria. Bacteroidetes is the dominant bacterial phylum in the rumen followed by Firmicutes and Proteobacteria [31,32]. However, the relative abundance of Firmicutes increases when the concentrate feed rate increases [33,34]. Therefore, the rumen fermentation conditions may be affected by growth stage. 

By collecting rumen fluid after the concentrated feed had been decomposed 2 h after feed intake, this study yielded a higher ratio of Firmicutes than previous studies. Henderson et al. [35] reported that *Prevotella*, *Butyrivibrio*, and *Ruminococcus* were dominant bacterial genera in the rumen of 35 livestock in 35 countries. *Prevotella* accounted for the highest proportion (31.6–42.6%) of all bacteria in this study, but no significant differences were observed among the growth stages (Table 5). *Metaprevotella* was significantly more abundant in G than in other growth stages (*p* < 0.05), whereas *Paraprevotella* tended to be more abundant in LF than in other growth stages (*p* = 0.056). *Paraprevotella* is an anaerobic microorganism that decomposes proteins and carbohydrates, and the high NFC in LF appears to be the cause of its higher abundance [36]. *Succiniclasticum* tended to be more abundant in EF than LF (*p* = 0.082). Significant differences were observed among *Christensenella*, *Gracilibacter*, and *Acetivibrio* in the growth stages, but these genera accounted for a low proportion of total bacteria (0.2–2.1%). 

Archaea were mainly represented by isolated species producing methane. *Methanobrevibacter* was dominant, accounting for 94.6–96.5% of total archaea. *Methanosphaera* tended to be more abundant in EF than in other growth stages (*p* = 0.091) but accounted for a low proportion of total archaea (0.5–1.5%). The phylum Neocallimastigomycota accounted for 64.1–84.0% of total fungi. The *Neocallimastix* ratio was high in G and EF compared to LF, whereas the *Orpinomyces* ratio was high in LF compared to the other growth stages. *Neocallimastix* is an obligate anaerobic fungus that degrades cellulose in the rumen [37]. Therefore, *Neocallimastix* was highly abundant in G and EF, which had a relatively high forage ratio. There was no difference in Protozoa among the different growth stages. The phylum Ciliophora accounted for 2.6–4.8% of total protozoa, but most of them (95.1–97.4%) were not accurately identified.

## 4. Conclusions

Growth stage was expected to affect rumen fermentation properties and microbial diversity in Hanwoo steers. We found differences in fermentation, such as related to those VFA productions. Moreover, certain bacterial differences were confirmed according to growth stage; however, there were no considerable changes in the major microbial genus in the rumen. In contrast, low-abundance microbial genera significantly differed between growth stages. Since various microorganisms coexist in the rumen and maintain their number within a certain range, there may be slight changes. Therefore, additional experiments are required to determine the cause of low-abundance microbial genera between the growth stages.

## Figures and Tables

**Table 1:** Chemical composition of experimental diets.

Items	G		EF		LF	
	Timothy	Concentrate	Grass Hay	Concentrate	Rice Straw	Concentrate
Dry matter (DM), %	94.3	91.9	95.6	96.8	95.3	96.8
% DM						
Crude protein (CP)	7.4	18.8	7.9	16.8	6.5	15.9
Ether extract	1.1	4.8	1.4	3.5	0.9	3.4
Non-fiber carbohydrate	20.7	32.5	24.5	52.2	23.6	49.5
Neutral detergent fiber	66.4	38.9	65.0	24.7	66.6	27.5
Acid detergent fiber	43.1	18.3	39.3	12.4	50.9	13.4
Crude ash	9.7	10.3	6.1	8.6	8.1	9.2
Neutral detergent insoluble CP	5.4	5.2	4.8	5.8	5.8	5.5
Acid detergent insoluble CP	1.2	1.5	1.5	1.0	2.0	1.1
Acid detergent lignin	5.7	5.5	5.0	3.2	7.1	4.1

G, growing stage; EF, early fattening stage; LF, late fattening stage.

**Table 2:** Primers for rumen microbial community.

Items	Primer	References
Bacteria	341F 5′-CCTACGGGNGGCWGCAG-3′ 805R 5′-GACTACHVGGGTATCTAATCC-3′	[22]
Archaea	915aF 5′-AGGAATTGGCGGGGGAGCAC-3′, 386R GCGGTGTGTGCAAGGAGC-3′	[23]
Fungi	MN100F 5′-TCCTACCCTTTGTGAATTTG-3′ MNGM2 5′-CTGCGTTCTTCATCGTTGCG-3′	[23]
Protozoa	841F 5′-GACTAGGGATTGGAGTGG-3′ 1302R 5′-AATTGCAAAGATCTATCCC-3′	[24,25]

**Table 3:** Comparison of rumen fermentation according to different growth stages.

Items	G	EF	LF	SEM	*p*-Value
pH	5.93	6.35	6.28	0.475	0.110
Ammonia nitrogen, mg/dL	23.97	21.83	19.12	1.866	0.148
Total volatile fatty acid, mM	106.96 ^b^	87.34 ^a^	70.02 ^a^	8.070	0.006
Acetate, %	61.06	57.68	57.82	4.532	0.276
Propionate, %	19.79 ^a^	21.19 ^b^	21.53 ^b^	1.605	0.019
Butyrate, %	16.82	16.13	15.46	1.284	0.537
Valerate, %	3.33 ^a^	5.02 ^b^	5.20 ^b^	0.425	0.000
Acetate to propionate ratio	3.05 ^b^	2.72 ^a^	2.69 ^a^	0.223	0.036

G, growing stage; EF, early fattening stage; LF, late fattening stage; SEM, standard error of mean. Means within a row not sharing the same letter (a, b) differ significantly (*p* < 0.05).

**Table 4:** Rumen microbial diversities from Hanwoo steers according to different growth stages.

Items	G	EF	LF
Bacteria			
OTUs	513.00	575.25	555.00
Chao1	640.36	682.77	683.27
Shannon	6.16	6.30	6.73
Gini-Simpson	0.95	0.95	0.98
Good’s Coverage	1.00	1.00	1.00
Archaea			
OTUs	63.25	65.00	69.75
Chao1	82.65	86.94	118.49
Shannon	1.23	1.46	1.73
Gini-Simpson	0.41	0.49	0.61
Good’s Coverage	1.00	1.00	1.00
Fungi			
OTUs	18.00	18.00	18.75
Chao1	18.25	18.00	19.00
Shannon	2.47	2.02	2.37
Gini-Simpson	0.79	0.71	0.75
Good’s Coverage	1.00	1.00	1.00
Protozoa			
OTUs	10.00	9.50	9.50
Chao1	10.25	10.13	10.25
Shannon	0.37	0.47	0.34
Gini-Simpson	0.11	0.14	0.10
Good’s Coverage	1.00	1.00	1.00

OUT, operational taxonomic unit; G, growing stage; EF, early fattening stage; LF, late fattening stage.

**Table 5:** Relative abundance of dominant taxa in Hanwoo steers according to different growth stages representing >0.1% of total sequences.

Phylum	Family	Genus	G	EF	LF
Bacteria					
Actinobacteria	Bifidobacteriaceae	*Bifidobacterium*	0.00	0.01	0.00
Bacteroidetes	Bacteroidaceae	*Bacteroides*	0.00	0.00	0.01
	Barnesiellaceae	*Barnesiella*	0.00	0.00	0.00
	Lentimicrobiaceae	*Lentimicrobium*	0.00	0.00	0.01
	Muribaculaceae	*Duncaniella*	0.03	0.02	0.02
		*Muribaculum*	0.00	0.00	0.00
	Paludibacteraceae	*Paludibacter*	0.02	0.03	0.04
	Porphyromonadaceae	*Porphyromonas*	0.00	0.00	0.00
	Prevotellaceae	*Marseilla*	0.00	0.00	0.00
		*Metaprevotella*	0.07 ^b^	0.01 ^a^	0.01 ^a^
		*Paraprevotella*	0.02 ^a^	0.02 ^a^	0.04 ^b^
		*Prevotella*	0.35	0.43	0.32
		*Prevotellamassilia*	0.00	0.00	0.00
	Tannerellaceae	*Parabacteroides*	0.00	0.00	0.01
	Flavobacteriaceae	*Capnocytophaga*	0.00	0.01	0.01
		*Galbibacter*	0.00	0.01	0.01
Fibrobacteres	Fibrobacteraceae	*Fibrobacter*	0.01	0.01	0.00
Firmicutes	Streptococcaceae	*Streptococcus*	0.00	0.01	0.01
	__	*Flintibacter*	0.01	0.01	0.01
	__	*Intestinimonas*	0.02	0.01	0.02
	Christensenellaceae	*hristensenella*	0.01 ^a^	0.01 ^a^	0.02 ^b^
	Clostridiaceae	*Falcatimonas*	0.00	0.00	0.00
	Eubacteriaceae	*Eubacterium*	0.00	0.01	0.01
	Gracilibacteraceae	*Gracilibacter*	0.01 ^b^	0.00 ^a^	0.01 ^ab^
	Lachnospiraceae	*Blautia*	0.00	0.00	0.00
		*Butyribacter*	0.00	0.00	0.00
		*Butyrivibrio*	0.02	0.01	0.02
		*Enterocloster*	0.01	0.01	0.01
		*Faecalicatena*	0.00	0.00	0.00
		*Lachnoanaerobaculum*	0.001	0.00	0.00
		*Lachnoclostridium*	0.00	0.00	0.00
		*Mediterraneibacter*	0.00	0.00	0.00
		*Novisyntrophococcus*	0.02	0.01	0.01
		*Pseudobutyrivibrio*	0.01	0.01	0.02
		*Tyzzerella*	0.00	0.0	0.0
	Oscillospiraceae	*__*	0.01 ^b^	0.00 ^a^	0.00 ^b^
		*Acetivibrio*	0.00 ^a^	0.00 ^ab^	0.01 ^b^
		*Anaerobacterium*	0.00	0.01	0.01
		*Ethanoligenens*	0.01	0.01	0.01
		*Ruminococcus*	0.03	0.03	0.04
		*Saccharofermentans*	0.01	0.01	0.01
		*Sporobacter*	0.00	0.00	0.00
	Coprobacillaceae	*Kandleria*	0.00	0.00	0.01
	Erysipelotrichaceae	*Erysipelothrix*	0.01	0.02	0.02
	Acidaminococcaceae	*Succiniclasticum*	0.04 ^ab^	0.06 ^b^	0.04 ^a^
	Selenomonadaceae	*Anaerovibrio*	0.01	0.01	0.01
		*Mitsuokella*	0.01	0.01	0.00
		*Schwartzia*	0.01	0.01	0.01
		*Selenomonas*	0.02	0.03	0.03
Proteobacteria	Kiloniellaceae	*Curvivirga*	0.00	0.01	0.00
	Rhodospirillaceae	*Rhodospirillum*	0.00	0.00	0.00
	Succinivibrionaceae	*Succinivibrio*	0.06	0.01	0.03
	Vibrionaceae	*Vibrio*	0.00	0.01	0.01
Spirochaetes	Treponemataceae	*Treponema*	0.00	0.00	0.00
Unclassified	Unclassified	unclassified	0.06	0.08	0.09
Archaea					
Candidatus Thermoplasmatota	Methanomassiliicoccaceae	*Methanomassiliicoccus*	0.00	0.00	0.00
Euryarchaeota	Methanobacteriaceae	*Methanobrevibacter*	0.96	0.95	0.97
		*Methanosphaera*	0.01 ^a^	0.02 ^b^	0.01 ^a^
Unclassified	unclassified	unclassified	0.02	0.02	0.01
Fungi					
Unassigned	other	other	0.00 ^a^	0.05^b^	0.00 ^a^
Other	other	other	0.06	0.02	0.02
Neocallimastigomycota	Neocallimastigaceae	other	0.23	0.29	0.14
		*Caecomyces*	0.15 ^b^	0.02 ^a^	0.12 ^b^
		*Neocallimastix*	0.51 ^b^	0.62 ^b^	0.23 ^a^
		*Orpinomyces*	0.06 ^a^	0.01 ^a^	0.48 ^b^
		*Piromyces*	0.00	0.00	0.01
		unclassified	0.00	0.00	0.00
Eukaryota					
Unclassified	unclassified	unclassified	0.95	0.97	0.95
Ciliophora	Ophryoscolecidae	*Diplodinium*	0.05	0.02	0.05
	Isotrichidae	*Isotricha*	0.00	0.00	0.00

G, growing stage; EF, early fattening stage; LF, late fattening stage. Means within a row not sharing the same letter (a, b) differ significantly (*p* < 0.05).

## Data Availability

The datasets used or analyzed during the current study are available from the corresponding author upon reasonable request.
